# Delaying gratification depends on social trust

**DOI:** 10.3389/fpsyg.2013.00355

**Published:** 2013-06-19

**Authors:** Laura Michaelson, Alejandro de la Vega, Christopher H. Chatham, Yuko Munakata

**Affiliations:** ^1^Department of Psychology and Neuroscience, University of ColoradoBoulder, CO, USA; ^2^Department of Cognitive, Linguistic, and Psychological Sciences, Brown UniversityProvidence, RI, USA

**Keywords:** cognitive processes, delay of gratification, decision making, intertemporal choice, social cognition

## Abstract

Delaying gratification is hard, yet predictive of important life outcomes, such as academic achievement and physical health. Prominent theories focus on the role of self-control, hypersensitivity to immediate rewards, and the cost of time spent waiting. However, delaying gratification may also require trust in people delivering future rewards as promised. To test the role of social trust, participants were presented with character vignettes and faces that varied in trustworthiness, and then choose between hypothetical smaller immediate or larger delayed rewards from those characters. Across two experiments, participants were less willing to wait for delayed rewards from less trustworthy characters, and perceived trustworthiness predicted willingness to delay gratification. These findings provide the first demonstration of a causal role for social trust in willingness to delay gratification, independent of other relevant factors, such as self-control or reward history. Thus, delaying gratification requires choosing not only a later reward, but a reward that is potentially less likely to be delivered, when there is doubt about the person promising it. Implications of this work include the need to revise prominent theories of delay of gratification, and new directions for interventions with populations characterized by impulsivity.

## Introduction

Delaying gratification is hard. Many people would rather enjoy a paycheck now than put money away for later. Everyone sometimes struggles to hold out for delayed rewards, but certain populations face particular difficulties, including addicts, criminals, obese individuals, depressed individuals, adolescents, and children (Wulfert et al., 2002; Hongwanishkul et al., 2005; Johnson et al., 2007; Anokhin et al., 2011; Casey et al., 2011). Moreover, the ability to delay gratification in childhood predicts important later life outcomes. For example, the ability to resist a desirable immediate treat in favor of a larger delayed one during preschool predicts higher SAT scores and better social competence in adolescence (Mischel et al., 1989; Shoda et al., 1990), and lower obesity rates and substance abuse in adulthood (Ayduk et al., 2000; Schlam et al., 2013). The tendency to treat future rewards as worth less than immediate rewards may therefore lead to undesirable consequences, both for the individual (e.g., lack of personal savings in case of emergency), and for society at large (e.g., insufficient long term investments in science and technology).

Prominent explanations of delaying gratification focus on the role of self-control, hypersensitivity to immediate rewards, and the cost of time spent waiting (Benzion et al., 1989; McClure et al., 2004; Zauberman and Lynch, 2005; Figner et al., 2010). However, delaying gratification also relies on the fundamental assumption that a future reward will be delivered as promised (e.g., that a portfolio manager will responsibly manage clients' savings; Frederick et al., 2002; Kidd et al., 2012; Mischel, 1961). That is, regardless of self-control, salience of immediate options, or perceived cost of time spent waiting, delaying gratification may only make sense when individuals believe that they would actually receive the delayed reward in the future if they opted to wait for it. Delaying gratification is not simply about choosing “more later” over “some now,” but rather, requires choosing “*maybe* more later” over “some now,” when there is doubt about whether those promising the future reward would come through.

Thus, delaying gratification may depend upon interpersonal trust, which refers to an interdependent relationship in which one party has a social expectation of cooperation from another party (Robinson, 1996; de Visser and Krueger, 2012). Trust in others is dynamically updated through experience (King-Casas et al., 2005), and can be can be modulated by information about others' prior behavior (Delgado et al., 2005) and perceptions of their ability to regulate their own behavior (Righetti and Finkenauer, 2011), as well as by motivational and affective states (Dunn and Schweitzer, 2005; Van den Bos et al., 2011). If delaying gratification depends upon expectations about an individual's likelihood of cooperation, variations in levels of trust should influence decisions to delay gratification.

Limited evidence is consistent with a role of social trust in delaying gratification, but is open to alternative interpretations. For example, children with fathers absent from the home (who might therefore be less inclined to trust others) are more likely to prefer smaller immediate rewards over larger delayed options (Mischel, 1961). Additionally, individuals who are less cooperative in a trust game also behave more impulsively in a temporal discounting task (Harris and Madden, 2002). However, such correlations could be driven by other factors, such as self-control, or by a causal relationship in the reverse direction, such that social cooperation requires the ability to delay gratification (Harris and Madden, 2002).

Some experimental work suggests a causal role of social trust in delaying gratification, but could alternatively be interpreted in terms of more general reward effects. For example, when rewards are promised by an experimenter but never provided, or are delivered inconsistently, preferences for immediate gratification increase in humans and other animals (Mahrer, 1956; Stevens et al., 2011; Kidd et al., 2012). This effect could arise from reduced trust that a delayed reward will be provided, but might alternatively arise from the changes to subjective well-being, motivation, and willpower that accompany reward provision/omission (Gomez and McLaren, 1997) and are known to influence self-control (Vansteenkiste and Deci, 2003; Ifcher and Zarghamee, 2011; Pyone and Isen, 2011; Lerner et al., 2013). That is, participants may have been less able to delay gratification because rewards that were inconsistent or withheld led to reduced self-control, rather than to reduced social trust.

The present studies thus test whether social trust, manipulated in the absence of rewards, influences choices about whether to delay gratification. We manipulated trust using vignettes about fictional characters (Experiment 1) and accompanying faces that varied in perceived trustworthiness (Experiment 2). We then assessed delay of gratification using a series of intertemporal choices in which participants were asked if they would prefer a smaller, immediate reward or a larger, delayed reward from the character they read about. We tested adults to build on prior manipulations of trust in the absence of rewards (Delgado et al., 2005; Oosterhof and Todorov, 2008; Fareri et al., 2012), and to obtain more precise estimates of willingness to delay.

## Experiment 1

All participants read three vignettes depicting trustworthy, untrustworthy, and neutral characters, then considered each character in delay of gratification situations.

### Materials and methods

#### Participants

Seventy-eight participants (34 male, 39 female, five who preferred not to indicate gender) between 18 and 64 years of age (*M*_age_ = 31.1 years, *SD* = 11.1 years) were paid $1.00 for completing the experiment, which lasted 10–15 min. Participants were recruited via Amazon's Mechanical Turk, a website that allows users to complete small tasks for pay, and had an average approval rating of at least 99% from previous jobs. Participants lived in the United States, and were normally distributed in terms of socioeconomic status, with the average participant having completed some college and receiving a financial income between $37,500–49,999 per year. All participants were included in the analyses; results were identical when excluding participants based on null discounting (i.e., all later responses in at least one condition, *N* = 2; as in Kirby and Maraković, 1996).

#### Materials and procedure

The experiment was presented in an online survey format. Participants first completed demographic questions. Then, as in Delgado et al. (2005), participants read the vignettes in a fixed order (trustworthy, untrustworthy, neutral) and completed trustworthiness ratings, using a scale of 1–7 to rate each individual on trustworthiness, likability, approachability, and likelihood of sharing. Next, participants completed intertemporal choice questions (as in Kirby and Maraković, 1996), which varied in immediate reward values ($15–83), delayed reward values ($30–85), and length of delays (10–75 days). Each question was modified to mention an individual from one of the vignettes [e.g., “If (trustworthy individual) offered you $40 now or $65 in 70 days, which would you choose?”]. Participants completed 63 questions in total, with 21 different questions that occurred once with each vignette, interleaved in a single fixed but random order for all participants. The 21 choices were classified into 7 ranks (using the classification system from Kirby and Maraković, 1996), where higher ranks should yield higher likelihood of delaying, allowing a rough estimation of a subject's willingness to delay using a small number of trials. Rewards were hypothetical, given that hypothetical and real rewards elicit equivalent behaviors (Madden et al., 2003) and brain activity (Bickel et al., 2009), and were preceded by instructions asking participants to consider each choice as if they would actually receive the option selected. Participants took as much time as they needed to complete the procedures.

### Results and discussion

Trust manipulated in the absence of reward, within subjects, influenced participants' willingness to delay gratification, with perceived trustworthiness predicting willingness to delay.

#### Approach and preliminary analyses

The effect of condition and rank on choice was analyzed with generalized linear mixed effect (lmer) models (with a logit link), using the lme4 package (Bates and Sarkar, 2006) in the R statistics package (R Development Core Team, 2006). Subjects' intercepts were modeled as random effects. This technique is a common alternative to ANOVA (e.g., Laubrock et al., 2007) and allowed us to model individual trial data to predict the probability of choosing the delayed option (“probability of delaying”) without averaging within individuals or rank. Validating the short temporal discounting assessment, the probability of delaying increased with rank, *b* = 0.81, *SE* = 0.15, *z* = 54.12, *p* < 0.001.

**Figure 1:**
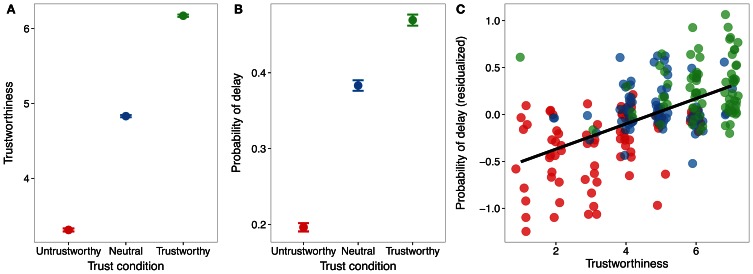
**(A)** Perceived trustworthiness increased as a function of trust condition. Error bars are standard error. **(B)** Probability of delaying gratification was lower in the untrustworthy condition (red) compared to the neutral (blue) and trustworthy conditions (green), reflecting reduced willingness to delay gratification with untrustworthy individuals. **(C)** Perceived trustworthiness correlates positively with probability of delay across conditions. Residuals after regressing out mean probability of delay for each subject is plotted on the y-axis. Individual data points are jittered 0.2 units on the x-axis for display purposes.

Perceived trustworthiness was predicted by condition (untrustworthy < neutral < trustworthy), *b* = 1.41, *SE* = 0.02, *t* = 90.9, *p* < 0.0001, suggesting our trust manipulation was effective (Figure 1A). The difference between untrustworthy and neutral conditions was not significantly different from the difference between neutral and trustworthy conditions, *b* = 0.18, *SE* = 0.27, *t* = 0.65, *p* > 0.51.

#### Effects of trust on delaying gratification

Participants' preference for delayed rewards, as indexed by probability of delaying, was predicted by condition, *b* = 0.76, *SE* = 0.04, *z* = 17.72, *p* < 0.0001; both untrustworthy (*b* = 1.48, *SE* = 0.23, *z* = 6.35, *p* < 0.001) and trustworthy (*b* = 0.49, *SE* = 0.08, *z* = 5.93, *p* < 0.001) conditions were significantly different from the neutral condition. In addition, the difference between untrustworthy and neutral conditions was significantly different from the difference between neutral and trustworthy conditions, *b* = 0.87, *SE* = 0.17, *t* = 5.18, *p* < 0.001, (Figure 1B); thus, our trust manipulation had a larger effect on delaying gratification at lower levels of trust, consistent with prior work showing non-linear effects of trust manipulations on other behaviors (Fareri et al., 2012). There was also an interaction between condition and rank, *b* = 0.11, *SE* = 0.02, *z* = 6.1, *p* < 0.001, such that the increase in delayed choices with rank was smaller in the untrustworthy condition relative to the trustworthy and neutral conditions. This suggests that as the delayed option became more appealing, those in the untrustworthy condition were more likely to continue to choose the immediate option. Importantly, perceived trustworthiness predicted probability of delaying, *b* = 0.49, *SE* = 0.03, *z* = 18.53 *p* < 0.0001, such that less perceived trustworthiness predicted lower willingness to delay gratification (Figure 1C). In addition, there was an interaction between condition and trustworthiness (*b* = 0.21, *SE* = 0.03, *z* = 6.30, *p* < 0.001), such that trustworthiness predicted probability of delaying within only the untrustworthy condition, consistent with a non-linear effect of trust on delay of gratification.

These results suggest that reducing social trust, in the absence of rewards, can decrease willingness to delay gratification. However, participants read all three vignettes and were asked to rate trustworthiness (to replicate Delgado et al., 2005) before making intertemporal choices, raising the possibility that they realized the study was investigating the role of trust in their choices, and responded based on their belief that trust should increase their willingness to delay. The fixed order of the vignettes also leaves open the possibility that perceived trustworthiness, willingness to delay, and their relationship were somehow driven by the order of vignettes. Experiment 2 addresses these issues by manipulating social trust between participants, and tests the replicability of the effects of social trust in the absence of rewards on delaying gratification.

## Experiment 2

All details were identical to Experiment 1 except where noted. Participants were randomly assigned to trustworthy, untrustworthy, or neutral conditions, rather than reading all three vignettes, and personality ratings were moved to the end of the survey, to minimize demand characteristics. To enhance the manipulation of social trust, each vignette was accompanied by a trustworthy, untrustworthy, or neutral computer-generated face. These faces were drawn from a larger database of faces manipulated to vary in trustworthiness (Oosterhof and Todorov, 2008) and known to influence trusting behavior (e.g., Oosterhof and Todorov, 2009; Todorov et al., 2009).

The between-subjects design of Experiment 2 allowed us to use a larger set of intertemporal choice questions, in a procedure similar to standard intertemporal choice tasks (Richards et al., 1999; Ballard and Knutson, 2009), so that we could calculate discounting rates (k-values). A much larger sample of participants was tested, to yield a more precise estimate of discounting rates and of the influence of our trust manipulation.

### Materials and methods

#### Participants

One hundred and seventy two participants (65 males, 60 females, 13 who preferred not to indicate gender) between 18 and 61 years of age (*M*_age_ = 28 years, *SD* = 8.9 years) participated in this study. Participants were paid $0.25 for completing this study, which took approximately 10 min. This lower pay rate was chosen given the larger sample size, and because compensation rates on Mechanical Turk only influence enrollment rate, not quality of the data (Buhrmester et al., 2011). All participants lived in the United States, and were normally distributed in terms of socioeconomic status, with the average participant having completed some college and receiving a financial income between $37,500–49,999 per year.

To maintain the between subjects design, we only included data collected on the first visit from any IP address; this resulted in the exclusion of 34 participants who completed surveys from more than one condition from the same IP address. All remaining participants were included in the analyses; results were identical when excluding subjects based on null or inconsistent temporal discounting behavior (as defined as in Johnson and Bickel, 2008, *N* = 22), or for completing the survey too quickly (<3 min, *N* = 3) as has been done in some studies (Lee, 2010; Bucholz and Latorre, 2011), but did not occur in Experiment 1. Final analyses included 46 participants in the trustworthy condition, 49 in the untrustworthy condition, and 43 in the neutral condition.

#### Materials and procedure

**Figure 2:**
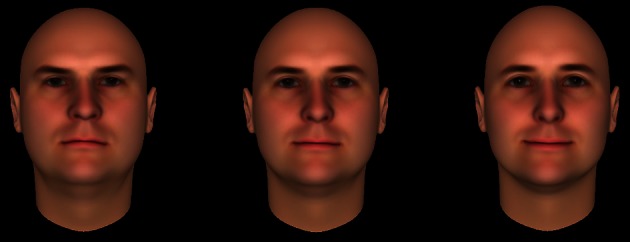
**Faces were paired with vignettes in Experiment 2, matching the condition to enhance the manipulation of trust**. Faces varied in trustworthiness from untrustworthy (left), neutral (middle), to trustworthy (right). A total of three different faces were used, each with untrustworthy, neutral, and trustworthy versions, yielding nine different faces overall.

Participants read one vignette, accompanied by a face. Three faces were selected from a database of 100 white male faces developed by Oosterhof and Todorov (2008) and implemented in the FaceGen Modeller program (Singular Inversions, Toronto, Ontario, Canada). Three faces were used to minimize effects of stimulus-specific variances related to the faces. Three variations—trustworthy, untrustworthy, and neutral—of each of the three faces were used, resulting in nine faces total (Figure 2). These variations differed in terms of characteristics that had been determined to be optimal in representing trustworthiness, based on principle component analysis of 300 emotionally neutral computerized faces that had been rated on a variety of social dimensions (Oosterhof and Todorov, 2008). For example, increasing the distance between the eyes and the eyebrows was associated with ratings of increased trustworthiness. Face images were 400 × 477 pixels in size.

Participants then completed the intertemporal choice task, which consisted of 49 binary choice questions between a smaller immediate reward ($5) and a larger delayed reward that varied in delay (4–150 days) and value ($11–34). The face from the vignette appeared three times across the intertemporal choice questions, to reinforce the character that the questions pertained to. Lastly, participants completed the personality ratings. Three participants had some missing rating scores and were excluded from rating analyses.

### Results and discussion

Trust manipulated in the absence of reward, between subjects, influenced participants' willingness to delay gratification, with perceived trustworthiness again predicting willingness to delay.

#### Approach and preliminary analyses

A *k* parameter was estimated for each participant (as in Ballard and Knutson, 2009), with lower *k*-values indicating increased preference for delayed rewards. Indifference points were calculated at each delay using logistic regression to determine the later value at which there was an equal probability of each response. When estimates were outside of the range of displayed later values (e.g., participants gave all later or now responses or gave inconsistent responses), indifference points were assumed to be just outside the range of values presented (34.5 for all “now” and 10.5 for all “later” responses). Discounted value (DV) was calculated at each delay (DV = $5/indifference point) and a hyperbolic discounting function was fit to all DVs using non-linear least squares: DV = 1/(1 + *k* × delay), where k is the unknown discounting parameter. As in previous research, this hyperbolic model provided a good fit for the data, as assessed using visual inspection and model comparison with an exponential function. There were no significant main effects or interactions with the different versions of trustworthy, untrustworthy, and neutral faces, so subsequent analyses collapse across faces within each trust condition. All analyses were completed using linear model (lm) in the R statistical package. All results were confirmed using bootstrapping, as k-values are not normally distributed.

**Figure 3:**
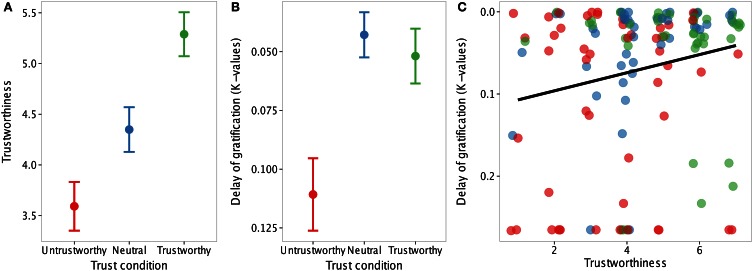
**(A)** Perceived trustworthiness increased as a function of trust condition. Error bars are standard error. **(B)** Discounting rates were higher in the untrustworthy condition (red) compared to the neutral (blue) and trustworthy conditions (green), reflecting reduced willingness to delay gratification with untrustworthy individuals. y-axis is reversed for conceptual consistency. **(C)** Perceived trustworthiness correlates positively with discounting rates. Correlation (95% confidence interval of *r*: 0.02–0.36) was verified using non-parametric bootstrapping due to positive skew in discounting values. Individual data points are jittered 0.2 units on the x-axis for display purposes.

Perceived trustworthiness was again predicted by condition (untrustworthy < neutral < trustworthy), *b* = 0.85, *SE* = 0.16, *t*_(130)_ = 5.34, *p* < 0.0001, Cohen's *d* = 0.45, suggesting that our trust manipulation was effective (Figure 3A). The difference between trustworthy and neutral conditions was not significantly different from the difference between neutral and untrustworthy conditions, as evidenced by overlapping 95% confidence intervals of parameter estimates for trustworthy-neutral (0.32, 1.52) and neutral-untrustworthy (0.12, 1.38).

#### Effects of trust on delaying gratification

Findings were largely consistent with Experiment 1. Participants' preferences for delayed rewards, as indexed by *k*, was predicted by condition, *b* = −0.03, *SE* = 0.01, *t*_(136)_ = −3.31, *p* < 0.005; participants were less willing to delay gratification in the untrustworthy condition than in the trustworthy and neutral conditions, *b* = 0.02, *SE* = 0.01, *t*_(136)_ = −4.13, *p* < 0.001, with no difference between trustworthy and neutral conditions, *b* = 0.01, *SE* = 0.02, *t*_(87)_ = 0.59, *p* = 0.55 (Figure 3B). The difference between untrustworthy and neutral conditions was greater than the difference between neutral and trustworthy conditions, as evidenced by non-overlapping 95% confidence intervals of parameter estimates for trustworthy-neutral (0.01, −0.02) and neutral-untrustworthy (0.03, 0.10); thus, as in Experiment 1, our trust manipulation had a larger effect on delaying gratification at lower levels of trust. The same pattern was observed across a model free, but less precise measure of delay of gratification: percentage of delayed choices across the experiment (trustworthy/neutral vs. untrustworthy: *b* = 0.07, *SE* = 0.2, *t*_(133)_ = 3.83, *p* < 0.001; trustworthy vs. neutral: *p* > 0.3). Finally, perceived trustworthiness predicted k-values (using non-parametric bootstrapping due to positive skew in discounting values, 95% CI: −0.001, −0.02, and using parametric analyses, *b* = −0.011, *SE* = 0.005, *t*_(135)_ = 2.4, *p* < 0.025), such that participants were less willing to delay gratification with characters perceived to be less trustworthy (Figure 3C).

## General discussion

Our results demonstrate that willingness to delay gratification depends on social trust. Whether contemplating a single interaction with one individual or multiple interactions with different individuals, people are less willing to wait for rewards with individuals they see as less trustworthy, when there is reason to doubt that an individual would actually deliver the delayed reward in the future. Directly experiencing the unreliability of an individual was unnecessary; here, impressions of trustworthiness from vignettes and faces produced powerful effects. This work complements prior correlational work, which suggested a link between trust and delaying gratification but did not establish causality (Mischel, 1961; Harris and Madden, 2002), and prior experimental work, which suggested that trust could influence delaying gratification but did not manipulate trust independent of rewards that can influence self-control (Mahrer, 1956; Kidd et al., 2012). Our studies provide the first experimental manipulation of trust while avoiding manipulations of reward, and thus critically demonstrate a causal role for social trust in delaying gratification, independent of other factors that can influence self-control.

Our findings add to a growing literature emphasizing the role of social factors in cognitive processes (Sanfey, 2007; Bernier et al., 2011; Meyer et al., 2012), and indicate the need to revise prominent theories of delay of gratification. Most theories focus on the role of cognitive control, basic valuation, and prospective mechanisms (Peters and Büchel, 2011). The role of social factors, while raised early on in this domain (Mischel, 1961), has been largely overlooked in subsequent theorizing and testing. For example, social factors go unmentioned in the burgeoning literature on the neural mechanisms supporting delay of gratification (Wittmann and Paulus, 2008; Luhmann, 2009; Peters and Büchel, 2011). However, our results demonstrate that delaying gratification does not occur in a social vacuum.

Our findings are also relevant to the study of social trust. For example, while existing studies have focused on the consequences of trust for immediate processing and behavior (McCabe et al., 2001; Delgado et al., 2005; Kosfeld et al., 2005; Van den Bos et al., 2011), the present work demonstrates how a lack of trust may also negatively impact planning for the future. In addition, in both experiments, we find non-linear effects of trust manipulations, like those observed in studies testing other effects of trust. For example, individuals invest less in the Trust Game with partners judged to be untrustworthy, but invest similarly with neutral and trustworthy partners (Fareri et al., 2012). In our experiments, participants were less willing to delay gratification with untrustworthy partners, and showed less differentiation between neutral and trustworthy partners. Such findings indicate a “threshold” for social trust, where decreases in trust below a certain threshold influence behavior more than increases in trust above that threshold, which seems worthy of further investigation.

Because our studies used trust vignettes and faces and hypothetical choices about whether to delay gratification, future work should build on these findings to examine the ability to actually wait for delayed gratification, with real people and rewards. Hypothetical and real rewards elicit similar patterns of temporal discounting behavior (Madden et al., 2003) and associated neural activity (Bickel et al., 2009), but social factors such as trust may matter more in situations involving an actual person rather than a vignette, or actual rewards rather than hypothetical ones. Similarly, hypothetical choices about delaying gratification and actually delaying gratification are correlated (Johnson and Bickel, 2002; Duckworth and Kern, 2011), but the influence of social factors may be more apparent when a hypothetical choice is first made rather than when a delayed choice must continue to be abided by (as in traditional delay of gratification paradigms), which may require additional processes, such as inhibitory control. Future studies could address such possibilities by comparing the influences of social trust on delaying gratification with vignettes vs. an experimenter (e.g., who behaves in a trustworthy or untrustworthy manner), with choices for immediate or delayed rewards vs. actually waiting for a delayed reward, and with hypothetical vs. real rewards (e.g., using primary rewards such as food, or randomly selecting and honoring one response from the intertemporal choice task; Reynolds et al., 2003).

Social factors suggest intriguing alternative interpretations of prior findings on delay of gratification, and suggest new directions for intervention. For example, the struggles of certain populations, such as addicts, criminals, and youth, might reflect their reduced ability to trust that rewards will be delivered as promised. Such variations in trust might reflect experience (e.g., children have little control over whether parents will provide a promised toy) and predisposition (e.g., with genetic variations predicting trust; Krueger et al., 2012). Children show little change in their ability to delay gratification across the 2–5 years age range (Beck et al., 2011), despite dramatic improvements in self-control, indicating that other factors must be at work. The fact that delay of gratification at 4-years predicts successful outcomes years or decades later (Casey et al., 2011; Shoda et al., 1990) might reflect the importance of delaying gratification in other processes, or the importance of individual differences in trust from an early age (e.g., Kidd et al., 2012). From this perspective, emphasizing social trustworthiness might be important in interventions for delaying gratification, not just for increasing accuracy of information collected from individuals with deficits (as emphasized for some interventions, e.g., with juvenile delinquents), but for improving behavior. Testing such possibilities for the role of social trust, and investigating how social and other factors interact, may greatly advance our understanding of the fundamental ability to delay gratification.

## Author contributions

All authors contributed to the development of the study hypothesis. Laura Michaelson conceived the study concept and designed the studies. Alejandro de la Vega performed the data analysis, with input from Christopher H. Chatham. Laura Michaelson, and Alejandro de la Vega drafted the manuscript, and Christopher H. Chatham and Yuko Munakata provided critical revisions. All authors discussed the results, implications, and literature, and approved the final version of the manuscript for submission.

### Conflict of interest statement

The authors declare that the research was conducted in the absence of any commercial or financial relationships that could be construed as a potential conflict of interest.
